# Immediate Hypersensitivity Reaction to Pheniramine in a Case of Multiple Drug Hypersensitivity Syndrome

**DOI:** 10.7759/cureus.79259

**Published:** 2025-02-18

**Authors:** Susamber Dik, Güzin Özden, Leyla Cevirme, Merve Erkoc, Hakan Basir

**Affiliations:** 1 Allergy and Immunology, Adana City Training and Research Hospital, Adana, TUR

**Keywords:** anaphylaxis, chronic urticaria, immediate type reaction, multiple drug hypersensitivity syndrome, pheniramine hydrogen maleate

## Abstract

Multiple drug hypersensitivity syndrome (MDHS) is a rare condition characterized by hypersensitivity reactions to at least two chemically unrelated drugs. While non-steroidal anti-inflammatory drugs and antibiotics are the most commonly implicated agents, hypersensitivity to antihistamines is extremely rare. A comprehensive approach, including detailed patient history, skin testing, and drug provocation tests, is essential for the accurate diagnosis and management of MDHS, particularly in patients with multiple drug reactions. When evaluating MDHS, antihistamines should also be considered as potential culprits if suggested by the patient’s history. Here, we aimed to present a case of a patient who reported allergic reactions following the administration of diclofenac sodium, paracetamol, COVID-19 mRNA vaccine, clarithromycin, and pheniramine hydrogen maleate. Based on the skin and provocation tests we performed, the patient was diagnosed with multiple drug hypersensitivity along with pheniramine allergy.

## Introduction

Multiple drug hypersensitivity syndrome (MDHS) is defined by allergic reactions to at least two chemically unrelated drugs [1]. Sullivan et al. initially described MDHS through case reports, establishing its definition based on clinical presentation and skin test results [2]. This syndrome is a distinct clinical entity from cross-reactivity, flare-up reactions, or multiple drug intolerance syndrome (MDIS) [1]. MDIS is defined as the occurrence of adverse drug reactions suggestive of a non-immunological mechanism to three or more drug classes in a patient [3].

In the majority of cases, the syndrome presents with acute urticaria, angioedema, or both following exposure to triggering compounds. Additionally, various cutaneous and systemic manifestations, such as Stevens-Johnson syndrome, anaphylaxis, serum sickness-like reactions, and immune cytopenias, have been documented [4].

A wide range of drugs has been implicated in MDHS, with antibiotics and non-steroidal anti-inflammatory drugs (NSAIDs) being the most commonly involved agents. Additionally, a broad spectrum of medications, including antiepileptics, opioids, angiotensin-converting enzyme inhibitors, corticosteroids, and psychotropic drugs, has also been associated with MDHS [3].

True MDHS is a rare condition, affecting 2.5% of patients with confirmed drug hypersensitivity reactions (DHRs) and 0.5% of patients presenting with self-reported DHRs [1].

Antihistamines are commonly used to manage symptoms associated with allergic reactions, insomnia, nausea, and viral infections. However, IgE-mediated type 1 hypersensitivity reactions due to antihistamines are exceedingly rare [5]. Type I hypersensitivity (immediate-type) reactions consist of sensitization and effector phases and involve the roles of Th2 cells, B cells, IgE, basophils, mast cells, and mediators such as histamine released from these cells [6].

The chemical structural diversity among antihistamine classes minimizes the risk of cross-reactivity [5]. Pheniramine maleate, a first-generation alkylamine-derived H1-antihistamine, is the parent compound of its group, which includes halogenated derivatives such as chlorpheniramine, brompheniramine, chlorpheniramine, and iodopheniramine [7]. Alkylamine-derived antihistamines may carry a risk of cross-reactivity due to structural similarities.

Reports of immediate hypersensitivity reactions due to pheniramine are exceptionally rare, even when cases of chlorpheniramine and dexchlorpheniramine hypersensitivity are included in the literature [8].

Here, we present a rare case of MDHS with confirmed hypersensitivity to pheniramine hydrogen maleate, an antihistamine frequently used in daily life for allergic reactions.

## Case presentation

A 37-year-old female patient presented to our clinic with generalized erythema, wheal, and dyspnea following drug administration. She had a five-to-six-year history of chronic urticaria and thyroid disease but no prior drug-related reactions. However, her first drug-induced reaction occurred 15 minutes after an intramuscular injection of diclofenac sodium. The patient developed generalized urticarial eruptions and dyspnea.

She also developed urticaria and dyspnea following the ingestion of paracetamol tablets and after receiving the COVID-19 mRNA vaccine. A similar reaction was observed after the administration of the clarithromycin tablet. After taking an unknown analgesic, she was admitted to the emergency department with tongue swelling. She was administered parenteral pheniramine hydrogen maleate, and within 15 minutes, widespread urticarial plaques developed.

As shown in Table 1, her laboratory test results were within the normal reference range. An alternative analgesic drug test was planned. The patient was administered a ¼ dose of an oral celecoxib tablet. Approximately 40 minutes later, erythema developed on her arms, abdomen, and neck. The oral provocation test (OPT) with celecoxib was considered positive. After this reaction, the serum tryptase level was measured at 2.98 ng/mL.

**Table 1:** Laboratory test results obtained after the initial examination and before drug testing

Parameter	Result	Reference range
White blood cell count	8.4	4.5-11 (10^3 µL)
Platelets	310	130-400 (10^3 µL)
Absolute eosinophil count	0.24	0-0.08 (10^3 µL)
Alanine aminotransferase	17	7-45 U/L
Aspartate aminotransferase	22	8-37 U/L
Thyroid-stimulating hormone	1.4	0.4-5.50 µIU/mL
Free thyroxine	1.24	0.8-1.72 ng/dL

**Figure 1:**
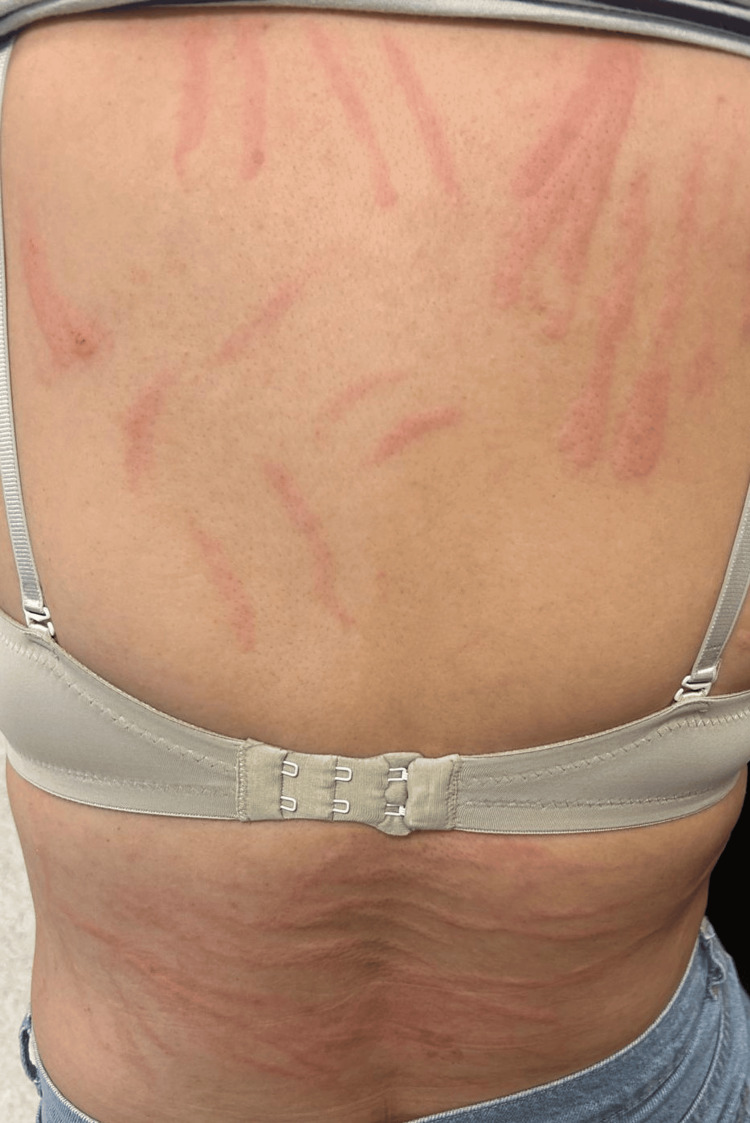
Linear urticaria after pheniramine hydrogen maleate infusion

Since the patient reported a suspected reaction to pheniramine hydrogen maleate, a skin prick and provocation test with pheniramine hydrogen maleate was planned. A skin prick test was performed with 45.5 mg/2 mL pheniramine hydrogen maleate at a 1:1 ratio, and an intradermal test was conducted with a 1:100 dilution. The skin tests yielded negative results. Subsequently, ¼ of 45.5 mg/2 mL pheniramine hydrogen maleate was diluted in 100 milliliters of isotonic NaCl solution and administered intravenously. After 10 minutes, widespread linear urticaria developed on the patient’s trunk (Figure 1). Informed consent was obtained from the patient.

The patient's basal serum tryptase level was 1.30 ng/mL, and the serum tryptase level following the pheniramine reaction was 3.14 ng/mL. The basal serum tryptase level increase was less than 20% + 2 ng/mL. Therefore, it was not classified as a severe anaphylactic reaction. When linear urticaria was observed, the pheniramine provocation test was considered positive.

As the description of the patient's reaction to clarithromycin was questionable in terms of hypersensitivity reaction, we planned a test with macrolides. To identify a safe antibiotic alternative, an OPT with dirithromycin was performed. Initially, one-quarter of a 250 mg dirithromycin tablet was administered for oral provocation. The patient was observed for 30 minutes, during which no reaction was noted. After this period, the remaining three-quarters of the tablets were administered. Approximately two hours after the last dose, linear urticaria appeared on the back and arms. However, the patient did not exhibit uvular edema, hypotension, or hypoxia. After each reaction, the patient was treated with 40 mg of intravenous methylprednisolone and oral cetirizine, leading to symptom resolution. To determine a safe antibiotic alternative, another drug test was planned with clarithromycin to confirm or exclude clarithromycin allergy. An OPT with clarithromycin was performed, yielding a negative result. Based on these findings, the patient was advised to avoid NSAIDs, pheniramine, and dirithromycin, while narcotic analgesics and clarithromycin were identified as safe alternatives.

## Discussion

This case was classified as MDHS due to hypersensitivity to three chemically distinct drug groups: antibiotics, NSAIDs, and antihistamines. Risk factors for MDHS include advanced age, female sex, chronic urticaria or angioedema, frequent hospitalizations, and multiple emergency room visits [3]. Our patient presented with two of these risk factors. As her reactions to antibiotics and analgesics were well-documented, further testing was unnecessary, and safe alternatives were identified. However, skin tests were conducted to confirm the allergy because her pheniramine-related reaction was uncertain. Multiple NSAID intolerance is generally recognized as a condition that predominantly occurs in patients with chronic urticaria. However, it can also be observed in individuals without a history of chronic urticaria [4]. Our case aligns with this clinical entity.

In recent years, there has been increasing emphasis on the role of skin and in vitro tests in diagnosing drug allergies. Although these tests cannot directly confirm causality, they provide evidence of sensitization and may have broader clinical applications due to their safety profile [8]. As chlorpheniramine and dexchlorpheniramine have been reported to be non-irritant at 1/100 dilution [8], we also used the maximum non-irritating dose of 1/100 dilution solution in our patient’s skin tests. In a case of generalized urticaria triggered by H1 antihistamines, skin prick tests were negative, whereas provocation tests were positive, suggesting that the reactions were not IgE mediated [9]. Negative skin prick and intradermal test results may be due to the low molecular weight of antihistamines, which act as haptens, or hypersensitivity to drug metabolites rather than the parent compound [10]. Since skin tests were negative, a drug provocation test was applied as the gold standard for diagnosis and was evaluated as positive upon reaction.

Although histamine is a key mediator in wheal and erythema formation, it is not the sole mediator. Other mediators, including platelet-derived growth factors, leukotrienes, and prostaglandins, also contribute to this response [5]. The urticaria observed following pheniramine provocation in our patient may be attributed to this mechanism. Cross-reactivity among antihistamines has not been extensively studied. Some cases of multiple antihistamine hypersensitivity have been reported, but most involved drugs do not share structural similarities [11]. The positive pheniramine provocation reaction was successfully treated with cetirizine, a piperazine-derived antihistamine with a different chemical structure.

The BAT result was negative in the first case in the literature, where a basophil activation test (BAT) was conducted for chlorpheniramine maleate. However, positive intradermal test results and the early onset of symptoms supported the likelihood of a type I hypersensitivity reaction [12]. Due to the unavailability of BAT in our region, it could not be performed. However, in our case, the positive result of the provocation test, the rapid onset of the reaction, and the mild increase in tryptase levels further support the likelihood of a type I (immediate-type) hypersensitivity reaction.

## Conclusions

In diagnosing drug hypersensitivity, a detailed history, considering the time of reaction onset, and, if necessary, skin and provocation testing are vital. The value of provocation tests in diagnosis has been demonstrated once again. In drug hypersensitivity, drug provocation testing remains the gold standard for evaluation. However, if accessible, BAT and drug-specific IgE tests should also be performed to support the diagnosis. Although it is a rare condition, antihistamine allergy should not be overlooked, even in cases where the clinical presentation is ambiguous. We recommend that antihistamines, along with their classifications, be considered in the differential diagnosis of drug hypersensitivity.
